# Proteomic and glycomic analyses of a lung-specific protein surfactant protein-D

**DOI:** 10.1016/j.dib.2015.09.017

**Published:** 2015-09-30

**Authors:** Emi Ito, Ritsuko Oka, Takeo Ishii, Hiroaki Korekane, Ayako Kurimoto, Yasuhiko Kizuka, Shinobu Kitazume, Shigeru Ariki, Motoko Takahashi, Yoshio Kuroki, Kozui Kida, Naoyuki Taniguchi

**Affiliations:** a Diease Glycomics Team, Systems Glycobiology Research Group, RIKEN-Max Planck Joint Research Center, RIKEN Global Research Cluster, 2-1 Hirosawa, Wako-shi, Saitama 351-0198, Japan; b Respiratory Care Clinic, Nippon Medical School, 4-7-15-8F Kudan-Minami, Chiyoda-ku, Tokyo 102-0074, Japan; c Department of Biochemistry, Sapporo Medical University School of Medicine, Sapporo 060-8556, Japan

## Abstract

In order to verify the protein enriched from pooled human sera to be a lung-specific protein surfactant protein-D (SP-D), we performed peptide mass fingerprinting (PMF)-based protein identification. MASCOT search results of the obtained PMF unequivocally demonstrated that it is identical to human SP-D. Meanwhile, we performed MALDI-QIT-TOF mass spectrometry-based *N*-glycomic analysis of the recombinant human SP-D produced in murine myeloma cells. The obtained mass spectra of *N*-glycans from the recombinant SP-D demonstrated that the recombinant protein is almost exclusively modified with core-fucosylated *N*-glycans [1].

**Specifications table**

**Table:** 

Subject area	*Chemistry*
More specific subject area	*Proteomics*
Type of data	*Figure*
How data was acquired	*Mass spectroscopy*
Data format	*Raw*
Experimental factors	*Not applicable*
Experimental features	*Proteomic identification of the enriched surfactant protein-D*
Data source location	*Disease Glycomics Team, Systems Glycobiology Research Group, RIKEN-Max Planck Joint Research Center, RIKEN Global Research Cluster, 2-1 Hirosawa, Wako-shi, Saitama 351-0198, Japan*
Data accessibility	*With this article*

**Value of the data**

• The enriched protein was verified to be surfactant protein-D (SP-D) by peptide mass fingerprinting.
• Any contaminated proteins other than SP-D were not identified by our proteomic analysis.
• A commercially available SP-D was found to be almost completely core-fucosylated by *N*-glycomic analysis.

## 1 Data, experimental design, materials and methods

This data article describes verification of the enriched SP-D from commercially available human pooled sera and results of *N*-glycomic analysis of a commercially available recombinant human SP-D produced in murine myeloma cells.

## 2 SP-D verification

**Fig. 1:**
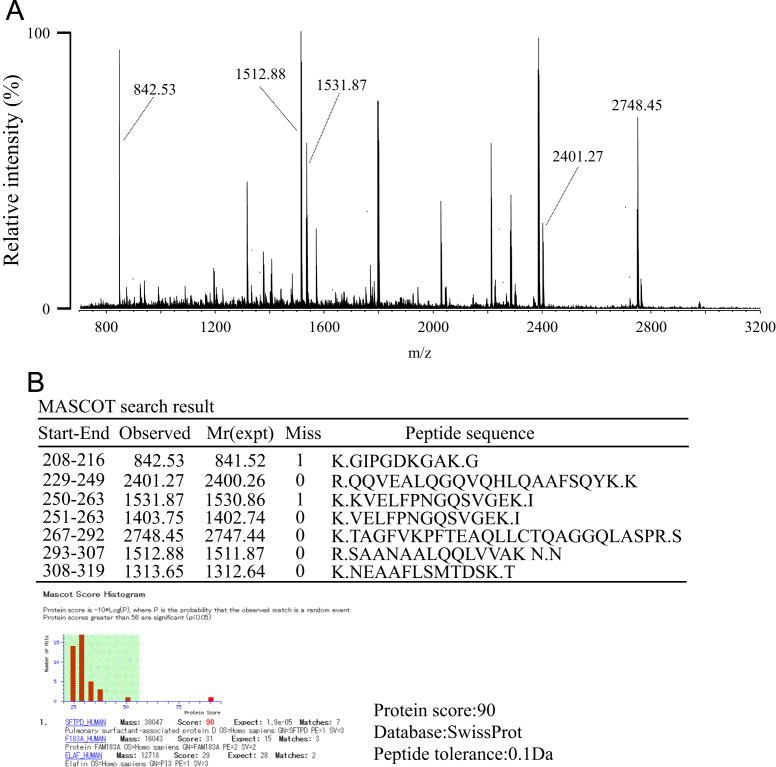
Protein identification of the SP-D-derived peptides. The SP-D enriched from pooled human sera was verified by proteomic analysis based on PMF. The obtained PMF data (A) were queried for protein identification using a Swiss-Prot database. The results unequivocally demonstrated that the queried protein is identical to human SP-D (B).

**Fig. 2:**
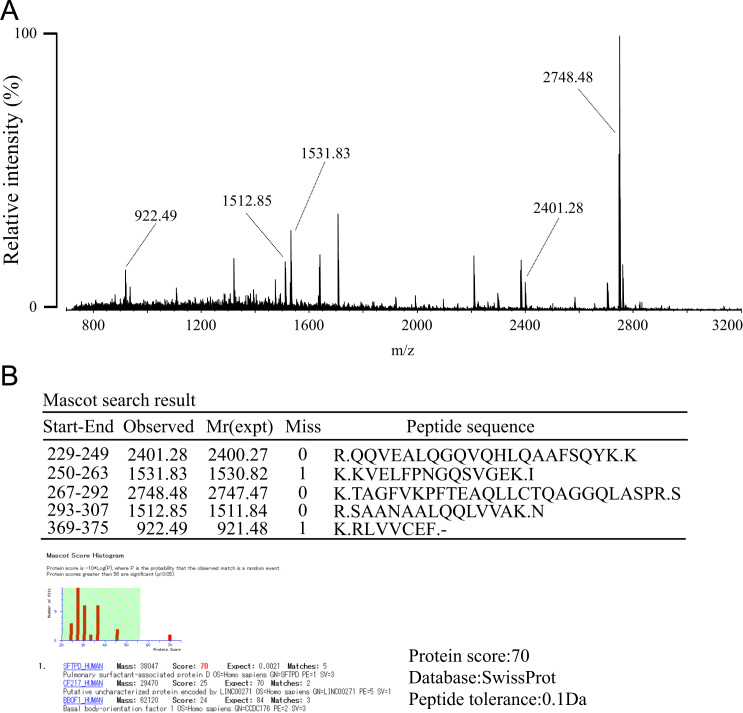
Protein identification of the SP-D-derived peptides. In the course of *N*-glycomic analysis of the enriched human serum SP-D [1], the protein portion of SP-D remained in a PAGE gel after liberation of *N*-glycans was verified by proteomic analysis based on PMF. The obtained PMF data (A) were queried for protein identification using a Swiss-Prot database. The results unequivocally demonstrated that the protein remained in a PAGE gel is human SP-D (B).

The verification of SP-D was performed by resolving the enriched SP-D by SDS-PAGE, in-gel digestion of the corresponding band, peptide mass fingerprinting, and MASCOT search. Briefly, human serum SP-D was enriched as described in our main research article [1]. The enriched SP-D fraction was resolved by SDS-PAGE under reducing conditions and visualized by CBB staining. The 43-kDa band was excised from the gel, cut into 1 mm^3^ pieces, and destained and dehydrated with 50% ACN/100 mM NH_4_HCO_3_. Proteins in the gel pieces were then reduced with 10 mM DTT in 100 mM NH_4_HCO_3_ at 56 °C for 1 h and alkylated with 55 mM IAA in 100 mM NH_4_HCO_3_ at room temperature in the dark for 45 min. The gel pieces were rinsed in 100 mM NH_4_HCO_3_ and dehydrated with 50% ACN/100 mM NH_4_HCO_3_, and then 100% ACN and then dried completely using a vacuum centrifuge concentrator. The dried gel pieces were rehydrated again with 10 ng/μl trypsin in 50 mM NH_4_HCO_3_ solution on ice, and then incubated at 37 °C for 16 h. The sample was heated at 90 °C for 5 min to inactivate trypsin and the resulting peptides were extracted with ACN/water/TFA (66:33:0.1, v/v/v). The extracts were then dried and desalted using a ZipTipμ-C_18_ tip (Millipore, Billerica, MA). The eluate (0.5 μl) and DHB matrix solution (0.5 μl) were deposited on a μFocus MALDI target plate (Hudson Surface Technology, Inc., Fort Lee, NJ) and left to dry at room temperature. All mass spectrometry was performed on a MALDI-QIT-TOF mass spectrometer (AXIMA-Resonance, Shimadzu/Kratos, U.K.). Ions were formed by a pulsed nitrogen UV laser (λ=337 nm) in both the positive and negative ion modes. A pulsed gas, He, was used for ion cooling in the ion trap and Ar was used for CID fragmentation. The DHB matrix solution was prepared by dissolving 2.5 mg of DHB in 1 ml of ACN/0.1% TFA in water (2:3, v/v). Protein identification was performed with MASCOT search (Matrix Science, London, U.K.) using the Swiss-Prot database 2014_12 by peptide mass fingerprinting with the following MASCOT parameters; miss cleavage: 2, peptide mass tolerance: 0.1 Da, taxonomy: human, fixed modifications: carbamidomethyl (C), variable modifications: oxidation (M), Pyro-Glu (N-term Q) (Figs. 1, 2).

## 3 *N*-Glycomic analysis of a recombinant SP-D

**Fig. 3:**
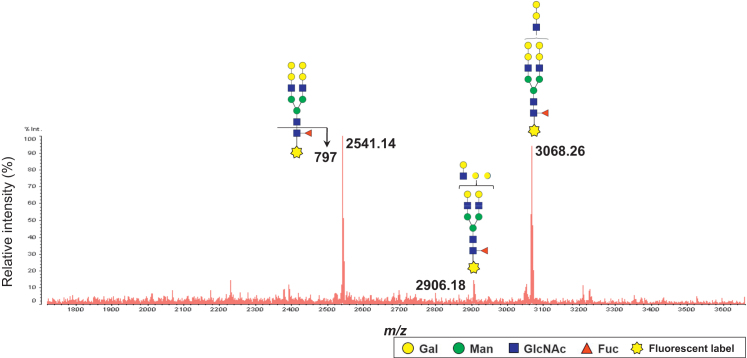
*N*-glycosylation profiling of recombinant human SP-D. *N*-Glycans in a commercially available recombinant human SP-D (R&D Systems) produced in murine myeloma N0 cells were released by digestion with PNGase F and the liberated *N*-glycans were labeled with BlotGlyco (Sumitomo Bakelite, Tokyo, Japan) according to the manufacturer’s instructions. The resulting fluorescence-labeled *N*-glycans were analyzed by MALDI-QIT-TOF mass spectrometry. The results unequivocally demonstrated the presence of a core-fucose in the recombinant SP-D *N*-glycans as revealed by confirming the characteristic fragment ion at *m/z* 797 (data not shown).

*N*-glycomic analysis of the recombinant SP-D was performed by liberating *N*-glycans with PNGase F from the protein portion, fluorescence labeling of the liberated *N*-glycans, and analyzing the resulting fluorescent *N*-glycans with a MALDI-QIT-TOF-mass spectrometer. Briefly, *N*-glycans in a commercially available recombinant human SP-D (R&D Systems) which was produced in murine myeloma N0 cells by manufacturer, were released by digestion with PNGase F and the liberated *N*-glycans were labeled with BlotGlyco (Sumitomo Bakelite, Tokyo, Japan) according to the manufacturer’s instructions. The resulting fluorescence-labeled glycans were then analyzed by MALDI-QIT-TOF mass spectrometry as described formerly (Fig. 3).
